# Ontogeny of osmoregulation of the Asian shore crab *Hemigrapsus sanguineus* at an invaded site of Europe

**DOI:** 10.1093/conphys/coab094

**Published:** 2021-12-31

**Authors:** Gabriela Torres, Guy Charmantier, Luis Giménez

**Affiliations:** Biologische Anstalt Helgoland, Alfred-Wegener-Institut, Helmholtz-Zentrum für Polar- und Meeresforschung, 27498 Helgoland, Germany; Marbec, Univ Montpellier, CNRS, Ifremer, IRD, 34095 cx 05 Montpellier, France; Biologische Anstalt Helgoland, Alfred-Wegener-Institut, Helmholtz-Zentrum für Polar- und Meeresforschung, 27498 Helgoland, Germany; School of Ocean Sciences, College of Environmental Sciences and Engineering, Bangor University, LL59 5AB Menai Bridge, UK

**Keywords:** shore crab, osmoregulation, ontogeny, larva, Invasive species

## Abstract

We studied the ontogeny of osmoregulation of the Asian shore crab *Hemigrapsus sanguineus* at an invaded area in the North Sea. *H. sanguineus* is native to Japan and China but has successfully invaded the Atlantic coast of North America and Europe. In the invaded areas, *H. sanguineus* is becoming a keystone species as driver of community structure and the adults compete with the shore crab *Carcinus maenas*. Strong osmoregulatory abilities may confer the potential to use and invade coastal areas already earlier in the life cycle. We reared larvae and first juveniles at 24°C in seawater from hatching to intermoult of each developmental stage (zoea I-V, megalopa, crab I). We exposed each stage to a range of salinities (0–39 ppt) for 24 h, and then we quantified haemolymph osmolality, using nano-osmometry. In addition, we quantified osmolality in field-collected adults after acclimation to the test salinities for 6 days. Larvae of *H. sanguineus* were able to hyper-osmoregulate at low salinities (15 and 20 ppt) over the complete larval development, although the capacity was reduced at the zoeal stage V; at higher salinities (25–39 ppt), all larval stages were osmoconformers. The capacity to slightly hypo-regulate at high salinity appeared in the first juvenile. Adults were able to hyper-osmoregulate at low salinities and hypo-regulate at concentrated seawater (39 ppt). *H. sanguineus* showed a strong capacity to osmoregulate as compared to its native competitor *C. maenas*, which only hyper-regulates at the first and last larval stages and does not hypo-regulate at the juvenile-adult stages. The capacity of *H. sanguineus* to osmoregulate over most of the life cycle should underpin the potential to invade empty niches in the coastal zone (characterized by low salinity and high temperatures). Osmoregulation abilities over the whole life cycle also constitute a strong competitive advantage over *C. maenas*.

## Introduction

Understanding and predicting the process of establishment of exotic species require better knowledge of the traits promoting invasion and colonization of new habitats. Physiological traits, as the foundation of environmental tolerance, are central to the process of invasion and species distribution (Ames *et al.*, 2020; Kelley, 2014). The capacity to osmoregulate is a key adaptation for the use of coastal and estuarine habitats (Péqueux, 1995; Charmantier, 1998; Anger, 2003). Osmoregulation (here referring to extracellular osmoregulation) is the capacity of an organism to actively regulate the osmotic pressure of the body fluids (Péqueux, 1995; Charmantier *et al*., 2009; Evans and Claiborne, 2009; Lignot and Charmantier, 2015; Rahi *et al*., 2018). Osmoregulation enables organisms to achieve optimal functioning over a range of salinities, maintain the concentration of essential substances, keep acid–base balance for the proper cell function (Whiteley, 2011; Henry *et al*., 2012; Whiteley and Taylor, 2015) and sustain growth and development (Torres *et al*., 2011). Osmoregulation is also likely to confer some capacity to tolerate ocean acidification conditions (Whiteley, 2011; Whiteley *et al*., 2018) and to facilitate evolutionary transitions from the marine to semi-terrestrial habitats (Anger and Charmantier, 2000).

Crustaceans are one of the most important groups of invaders in coastal areas (Galil *et al*., 2011). Tolerance to low salinity is a key trait within the most notable crustacean invaders (Rudnick *et al*., 2005; Roche *et al*., 2009; Fowler *et al*., 2013); low, high or variable salinities are characteristic of intertidal zones and estuaries. In invasive crustaceans, the ontogeny of osmoregulation is likely to influence the process of invasion through changes in the so-called ‘propagule pressure’ (*sensu*  Simberloff, 2009). Little propagule pressure towards coastal-estuarine habitats would be expected from marine crustaceans, as they generally are stenohaline and weak osmoregulators or osmoconformers over their full life cycle (i.e. the osmolality of their body fluids is roughly equal to that of the surrounding fluid: Péqueux, 1995; Charmantier *et al*., 2009). By contrast, invasive estuarine species are osmoregulators at least during the adult stage (Rudnick *et al*., 2005; Fowler *et al*., 2013), but the full process of invasion may also depend on the life-history strategy during the larval phase. Estuarine species or those living in land-locked habitats show a pattern of osmoregulation that varies according to the strategy of ontogenetic migration. For instance, species retaining their larvae in the parental habitat (e.g. within estuaries or other land-locked water masses) are able to osmoregulate over the full life cycle (Charmantier *et al*., 1998; Anger and Charmantier, 2000, 2011). Therefore, for an invasive species following a larval retention strategy, invasion could theoretically take place at any stage of development. By contrast, the process of invasion will be restricted to specific life stages in species following the larval export strategy, i.e. where larvae are exported to coastal waters, characterized by higher and more stable salinities. In such species, the osmoregulatory capacity (OC), and hence the ability to use estuarine waters as habitat, is reduced or absent during the osmoconforming larval stages (Charmantier *et al*., 2002; Cieluch *et al*., 2004; Anger *et al*., 2008).

The ontogeny of osmoregulation should also drive the capacity of an exotic species to establish a self-sustaining population in a new habitat. Self-sustaining populations are a key for the long-term establishment, as well as for range expansion, a major characteristic of invasions (Gurevitch *et al*., 2011). Theoretically, self-sustaining populations can play a role in sustaining sink populations at the range limit (Dauphinais *et al*., 2018; Giménez *et al*., 2020). In species showing larval export strategy, successful establishment of local populations must rely in the capacity of larvae to perform ontogenetic migrations from and to the parental habitats. However, such capacity can vary regionally depending on hydrodynamic processes driving larval transport (Schab *et al*., 2013; Shanks *et al*., 2017), as well as the timing of reacquisition of the capacity to osmoregulate (Torres *et al*., 2006). By contrast, in species that are able to osmoregulate over the full life cycle, the establishment of local self-sustaining populations should not be restricted to specific life phases.

Here we report on the ontogeny of osmoregulation of the Asian shore crab *Hemigrapsus sanguineus*. *H. sanguineus* is native to the Pacific coast (Japan, China and Russia; Stephenson *et al*., 2009), but over the past 50 years, it has invaded the Atlantic coasts of North America and North Europe. In North America, *H. sanguineus* was first recorded in the 1980s, in the Delaware Bay and it subsequently expanded northwards over 10 degrees of latitude (Epifanio *et al*., 1998; Stephenson *et al*., 2009; Epifanio, 2013; Lord and Williams, 2017). In Europe, *H. sanguineus* was first found in the Dutch delta system in 1999 (Dauvin *et al*., 2009) and then it expanded over the North Sea (Jungblut *et al*., 2017; Geburzi *et al*., 2018; Jungblut *et al*., 2018), reaching also the coast of Scandinavia (Karlsson *et al*., 2019). In the German Bight (North Sea), *H. sanguineus* has invaded the intertidal zones of the Wadden Sea where densities average reached values of the order of 500 crabs m^−2^ (Geburzi *et al*., 2018; Fig 3, C6: size range ≤ 10 mm).

The impact of *H. sanguineus* on other species, including mussels and crabs, has been recorded in both the Atlantic coast of USA and in N. Europe (Stephenson *et al*., 2009). As a mode of comparison, in the Atlantic coast of USA, the increase in abundance of *H. sanguineus* correlates with the disappearance of another invasive species, the shore crab *Carcinus maenas* (Lohrer and Whitlatch, 2002), which has invaded coast worldwide (Carlton and Cohen, 2003; Roman and Palumbi, 2004) and is included in the list of the top 100 alien invasive species in the world (Global Invasive Species Database, 2021). In the US coast, adults of *H. sanguineus* outcompete *C. maenas* through predation on juveniles and shifts in diet that ultimately drive fecundity (Jensen *et al*., 2002; Griffen *et al*., 2011). We know less about how those species compare at their dispersive larval phase, a key trait driving invasions (Hassall *et al*., 2008; Simberloff, 2009).

*H. sanguineus* is a euryhaline crab with larvae capable to withstand salinities as low as 15 ppt (Epifanio *et al*., 1998), although the megalopa appears to exhibit reduced salinity tolerance. In the present study, we quantified the ontogeny of the capacity to osmoregulate in the five zoeal stages and the megalopa, in the first crab (juvenile) stage, and in adults, thereby covering the full larval and benthic life phases. We studied osmoregulation at the optimal temperature in order to quantify the full osmoregulatory potential of the species. The range of salinities used are those to be experienced by *H. sanguineus* populations in North Europe and associated estuarine areas (Karlsson *et al*., 2019), including the mouth of the Elbe and Weser rivers in the German Bight (surface salinities 15–32 ppt: Bils *et al*., 2012; Sprong *et al*., 2020), Dutch delta system (salinities range 12–32 ppt in the Osterschelde estuary: Gerringa *et al*., 1998), Kattegat (salinity 18–34 ppt). Moderately low salinities may be experienced by populations located in North America (e.g. mouth of Delaware Bay) and in the Western Pacific coast (range distribution in Fig. 9 of Stephenson *et al*., 2009). We also tested osmoregulation at high salinities (>35 ppt) because salinity can increase above that of seawater in intertidal pools in the summer. We show that *H. sanguineus* has the capacity to osmoregulate over the full life cycle, an adaptation that is likely to underpin the invasion capacity, enhancing the dispersal ability and the capacity to deal with competitors such as *C. maenas*.

## Materials and Methods

### Collection of adults and larval rearing of *H. sanguineus* (De Haan, 1835)

Experiments were carried out with adults and larvae from the local population of Helgoland and Sylt (North Sea, German Bight) during the reproductive period. Egg-carrying females were collected in intertidal habitats and kept in the Helgoland laboratory in 2 L aquaria with oxygenated and filtered (0.2 μm) natural seawater (32.5 ppt). Aquaria were placed in a temperature-controlled room at 24°C with a 12:12 h light:dark cycle.

Experiments with adults were based on field-collected individuals. Experiments with larvae and first stage juveniles were run using standard methods of larval rearing (Charmantier *et al*., 2002; Torres *et al*., 2020, 2021b). Larvae were reared in filtered aerated natural seawater in groups of 50 individuals in 500 ml glass bowls (density: 0.1 larva ml^−1^) in a temperature-controlled room at 24°C, 12:12 h light:dark cycle. Daily, rearing bowls were rinsed and cleaned, water was changed, dead individuals were removed and larvae were fed with freshly hatched *Artemia* sp. nauplii. Prior to experiments, larvae moulting to each stage were separated daily from the cultures and kept in additional bowls in order to obtain larvae with the same moulting age. Survival was tested at each stage at all salinities. Animals were checked at regular intervals and determined as dead when not moving after being repeatedly touched with a probe.

### Experiments

Osmolality was adjusted with a salinometer (Cond 3110 SET1, WTW GmbH, Weilheim, Germany) by diluting natural seawater (32.5 ppt = 966 ± 1 mOsm kg^−1^) with appropriate amounts of tap water. Water (samples of 20 μl) was then checked to achieve the appropriate osmolality using a micro-osmometer (Model 3MO, Advanced Instruments, Needham Heights, MA, USA); conversion factors between osmolality and salinity are as follows: 3.36 ppt ≈ 100 mOsm kg^−1^; 29.7 mOsm kg^−1^ ≈ 1 ppt.

**Figure 1:**
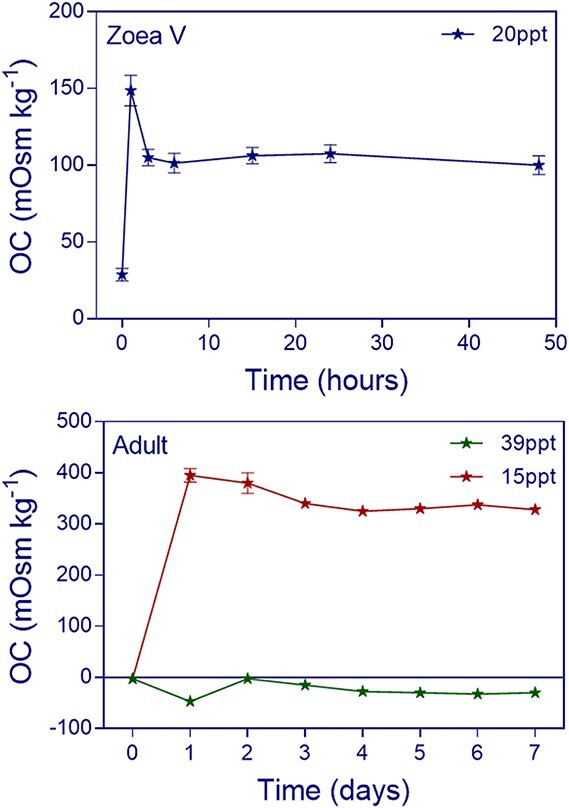
*Hemigrapsus sanguineus*. OC change through time. Haemolymph osmolality was measured in zoea V (upper panel) and adults (bottom panel) after transfer from seawater (32.5 ppt) to 20 ppt (blue) and to 15 ppt (red) and 39 ppt (green), respectively. Values are shown as means ± standard error (*n* = 6–8 for Z V; *n* = 3–5 for adults for each measurement).

Haemolymph osmolality was quantified using nano-osmometry (Charmantier *et al*., 1998); OC was calculated as the difference between the haemolymph and medium osmolalities. OC was quantified at intermoult; freshly hatched or recently moulted larvae or juveniles were separated from cultures and kept in vessels for 50% of the expected stage duration when they were checked visually (based on preliminary experiments). At the appropriate time, larvae of each stage were placed in petri dishes at the appropriate test salinities and kept for 24 h (see Fig. 1 hereafter). Before proceeding with the measurements, larvae were quickly rinsed in deionized water and gently dried on a filter paper. They were then submersed in mineral oil in order to avoid evaporation and desiccation (any remaining water was aspired using a first micropipette). Samples of haemolymph (sample volume ~ 30 nl) were taken from the heart by inserting a second micropipette into the body. Adults were placed in individual aquaria and maintained in the test salinities. Prior to haemolymph sampling, their moulting stage was checked through microscopic examination of pleopod setae (Drach, 1939; Drach and Tchernigovtzeff, 1967) and only crabs in intermoult stage C were retained. After 6 days of exposure (see Fig. 1 hereafter), the crabs were rinsed with deionized water and dried with filter paper. Haemolymph was sampled with a micropipette inserted at the basis of a posterior pereiopod, and it was quickly transferred into mineral oil to avoid evaporation. For all experimental stages, haemolymph osmolality was determined with reference to the medium osmolality on a Kalber–Clifton nanoliter osmometer (Clifton Technical Physics, Hartford, NY, USA).

### Data analysis

For the range of osmolalities between 15 and 39 ppt, data of OC by stage was analysed through two-way factorial analysis of variance (ANOVA) (Underwood, 1997) after confirmation that variance did not show evidence of heterogeneity (Cochran test, *P* = 0.38) and residuals showed normal distribution (qq-plot of within cell residuals). Both the first juvenile (C I) and adults survived at lower salinities than the larval stages, but we did not record measurements for the C I in seawater. Therefore, the comparison of osmoregulatory capacities was carried out through separate ANOVAs considering (i) megalopa and first juvenile, and (ii) first juvenile *vs*. adult. In addition, for adult crabs, we also considered responses under pure freshwater (i.e. 0.03 ppt, shown as < 1 ppt in Fig. 3) and 10 ppt, which were analysed along with the responses at the remaining osmolalities using one-way ANOVA. Differences among treatment combinations were determined using the Student–Newmann–Keuls test.

### Results

The **percentage survival** after exposure to the test salinities for 24 h or 6 days for larvae and juvenile or adults, respectively, is given in Table 1. Recall that different time periods between adults and larvae were needed to achieve stability for measurements of osmoregulation. Differences between those time periods do not explain the contrasting differences in survival rates between larvae and adults. Larvae did not survive at 10 ppt or lower salinities while survival was 60–100% at 20 ppt or higher salinities. Advanced zoeal stages and the megalopa showed moderate to low survival rates at 15 ppt. However, adults survived in all tested salinities until 6 days, even if there was only one survivor in freshwater (< 1 ppt). Hence, comparisons based on instantaneous mortality rates would show even a stronger contrast between larval and adult mortality at different salinities.

**Table 1:** *Hemigrapsus sanguineus*: Percent survival of the tested stages according to the different salinities, after exposing each larval stage and the first juvenile for 24 h, and the adults for 6–7 days

Osmolality (mOsm kg^−1^)	1	145	290	434	581	730	942	1146
Salinity (ppt)	**<1**	**5**	**10**	**15**	**20**	**25**	**32.5**	**38.7**
ZI	0	0	0	100	100	100	100	100
ZII	0	0	0	100	100	100	100	90
ZIII	0	0	0	33	100	100	90	100
ZIV	0	0	0	33	100	100	100	100
ZV	0	0	0	25	100	100	100	100
M	0	0	0	30	80	70	60	60
CI	NT	NT	100	100	80	100	NT	100
Adults	5	20	60	100	100	90	90	75

Note: The number of individuals at the start of the exposure was 10 for all larval stages and 5 for juveniles (in the tested salinities); except at 15 ppt for zoea III and IV (*n* = 15), zoea V and megalopa (*n* = 20). The number of adults at the start of the exposure was 10 for all salinities, except at freshwater (*n* = 20), 5 ppt (*n* = 15) and 15 and 38.7 ppt (*n* = 12). Survival of adults was 100% at all salinities except at < 1 and 5 ppt: 5 and 20%, respectively (i.e. animals that survived the first 24 h, also survived until the osmoregulation measurements were performed). NT: not tested (due to low number of individuals reaching CI, we prioritized the other salinities; indicated with an asterisk in Fig. 3).

The **acclimation time** after an abrupt change in salinity was determined in zoea V as a representative for the larval stages, and in adults, using for each of these stages a salinity for which survival was high. We determined the acclimation time for zoea V and adults at the selected salinity (a salinity for which survival was high: preliminary experiments). For zoea V, such tests showed that haemolymph osmolality and OC stabilized after less than 10 hours of exposure; for adults, stabilization occurred after ca. 4–5 days depending on salinity (Fig. 1). The period of exposure of larvae (24 h) and adults (6 days) to the different salinities was established after these tests and was used for the subsequent experiments.

**Figure 2:**
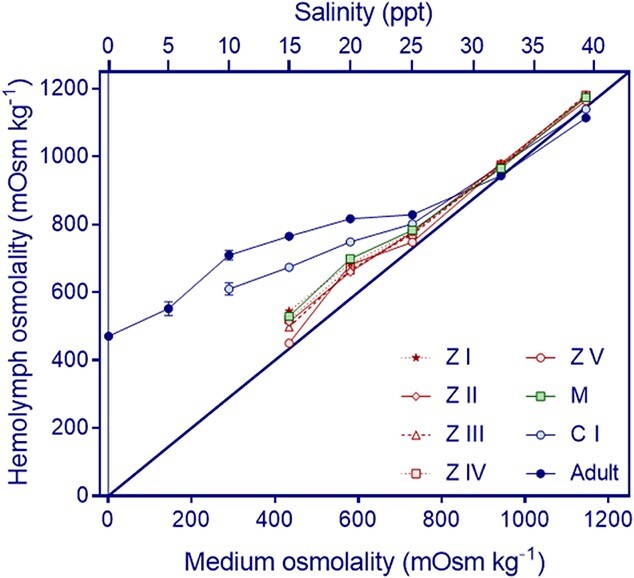
*Hemigrapsus sanguineus*. Variations in the haemolymph osmolality in different life cycle stages in relation to the osmolality (bottom *x* axis) and salinity (upper *x* axis) of the medium at 24°C. Acclimation time was 24 h for larval and crab I stages, and 6 days for adults. Values are shown as average values ± standard error. For zoeal stages I–III: *n* = 9–10, IV–V: *n* = 5; for megalopa: *n* = 6–8, for crab I: *n* = 4–5; for adults: *n* = 9–12, except for crabs exposed to < 1 ppt: *n* = 1, 5 ppt: *n* = 3 and 10 ppt: *n* = 6. Zoeal stages are shown in red (Z I: stars, Z II: diamonds, Z III: triangles, Z IV: squares and Z V: circles); megalopa (M) is shown in green squares; first juvenile crab (C I) is shown in light blue circles and adult is shown in dark blue circles. Note that standard errors may be smaller than symbols.

**Figure 3:**
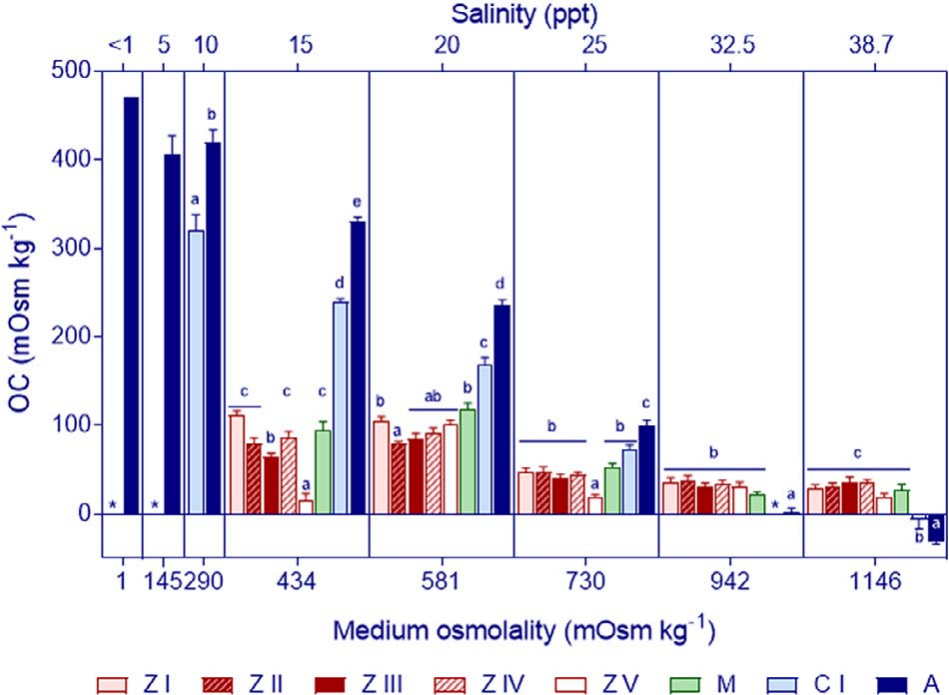
*Hemigrapsus sanguineus*. Variations in OC at different life cycle stages in relation to the osmolality (bottom *x* axis) and salinity (upper *x* axis) of the medium at 24°C. Values are shown as average values ± standard error (replicate numbers as in Fig. 2). Zoeal stages (ZI–ZV): red bars; megalopa (M): green bars; Juvenile I crabs (CI): light blue bars; adults crabs (A): dark blue bars; missing bars at < 15 ppt (434 mOsm kg^−1^) denote 100% mortality in all larval stages; asterisks instead of bars show salinities not tested for CI.

The **ability to osmoregulate** is illustrated by variations of haemolymph osmolality (Fig. 2) and of OC (Fig. 3) according to salinity for each tested stage. These results revealed significant variations with salinity showing a pattern consistent with hyper- and hypo-regulation (Fig. 2), but such capacity varied considerably among stages (significant interaction stage by salinity: Fig. 3 and Table 2). The zoea I-IV and the megalopa were able to hyper-regulate within the range of 434–581 mOsm kg^−1^ (15 to 25 ppt) and hyper-osmoconformed in the range of 730–1146 mOsm kg^−1^ (25 to 38.7 ppt). The zoea V, by contrast showed a weak capacity to osmoregulate, evident only at 581 (20 ppt).

**Table 2:** *Hemigrapsus sanguineus*: Analysis of variance evaluating the effect of salinity and stage in the haemolymph osmolality during the life cycle

	*df*	MS	F	P
Stage (S)	6	38 955	163	<10^−4^
Osmolality (O)	4	125 477	526	<10^−4^
S:O	24	24 765	104	<10^−4^
Residual	280	239		

This analysis considered all zoeal stages, the megalopa and the adult for salinities ranging between 15 ppt (= 434 mOsm kg^−1^) and 39 ppt (= 1146 mOsm kg^−1^). Abbreviations: *df*: degrees of freedom; MS: mean squares, F: Fisher statistics, *P*: *P* value.

First juvenile crabs (C I) differed in their OC with respect to the megalopa and adults but the difference depended on salinity (Stage:Salinity: comparison with megalopa: *F*_3,38_ = 53.5, *P* < 10^−12^; comparison with adult: *F*_4,60_ = 19.8, *P* < 10^−9^). At 15 and 20 ppt, juveniles were stronger hyper-regulators than the megalopa while they did not differ significantly in osmoregulation at 25 ppt; in addition, at 39 ppt, juveniles showed on average a weak capacity to hypo-regulate. When compared to adults, juveniles were weaker hyper-regulators. In addition, at 39 ppt the OC was not significantly different from zero: two out of 5 crab I juveniles had positive values of OC (20 and 40) while other two had negative values.

Adults had comparatively the strongest OC of all tested stages (Fig. 3), and, at least 5% managed to survive in freshwater (<1 ppt), while 20–60% survived at 5 and 10 ppt, respectively (Table 1). They hyper-osmoregulated in the range 434–730 mOsm kg^−1^ where the OC increased three times with respect to the larval stages. Adults also hypo-osmoregulated at 1146 mOsm kg^−1^ where survival was 75% (Table 1).

## Discussion

We found that *Hemigrapsus sanguineus* was able to osmoregulate over the entire life cycle, a trait that is likely to contribute to the invasion potential. To our knowledge, there is no native crab species in the North Sea with the capacity to osmoregulate across the full life cycle. Such capacity is not present in larvae of *C. maenas* (Cieluch *et al*., 2004, Torres *et al.*, 2021a), the most important native crab occupying intertidal and estuarine zones of North Europe and in addition to being a global invader (Carlton and Cohen, 2003; Roman and Palumbi, 2004). The osmoregulatory pattern of *H. sanguineus* is only comparable to the one exhibited by larvae of *Eriocheir sinensis* (Cieluch *et al*., 2007), another invasive crab found in the North European Seas (Ojaveer *et al*., 2007). The crab *E. sinensis* is also included in the top 100 invasive alien species of the world (Global Invasive Species Database, 2021). Hence, given the realized invasion of *E. sinensis*, which includes the Baltic Sea, the osmoregulatory pattern reported in this paper is indicative of the strong potential of *H. sanguineus* to establish coastal populations in the newly invaded areas. Whether such full potential is realized over the full life cycle will depend on other factors, such as temperature, which is known to modulate OC in crustaceans (Torres *et al*., 2021b) and drive salinity tolerance in the larvae of *H. sanguineus* (Epifanio *et al*., 1998). Hence, whether the full potential is currently realized will depend on latitude, with those local populations present at lower latitude being nearer such potential. However, the realized potential is likely to increase in the future, especially in the Channel and Southern North Seas where isotherms are shifting at a speed of 100 km per decade (Burrows *et al*., 2011), temperatures have increased at a rate of ~ 0.4–0.8°C per decade (Huthnance *et al*., 2016) and are expected to increase another 2–3°C by 2100 (Schrum *et al*., 2016). Our data on acute exposure (Table 1) and those of Epifanio *et al*. (1998) on chronic exposure suggest that larvae of *H. sanguineus* may close the life cycle in habitats of moderately low salinity (> 15 ppt) in situations where larvae experience temperatures in the range of 18–25°C. Adults show a much wider thermal tolerance as shown for instance by populations in the Wadden Sea where winter temperature drops to < 10°C.

Adults of *H. sanguineus* showed a strong capacity to hyper-regulate in diluted seawater over a range of salinities (0–20 ppt) although survival rates at the lowest tested media was low (Table 1). Our results confirm the hyper-regulatory pattern in our exotic local populations (Sylt and Helgoland, North Sea), as well as the time adults needed to achieve full osmoregulation (> 4 days), found by Watanabe (1982) for a native population (Hokkaido, Japan) and by Hudson *et al*. (2018) for a population of North America (Connecticut, USA). The capacity to hyper-regulate in *H. sanguineus* (maximum of ~ 400–470 mOsm kg^−1^ at 5 ppt and in fresh water was lower than that of strong osmoregulators such as *Armases miersii* and *Neohelice granulata* (up to 600 mOsm kg^−1^ at 1 ppt: Charmantier *et al*., 1998; Charmantier *et al*., 2002), which occur respectively in rocky intertidal and estuaries pools.

We also found in the adult a significant capacity to hypo-regulate at high salinities (38.5 ppt). We do not have strong evidence showing that the capacity to hypo-osmoregulate is already present at the first juvenile stage; slight hypo-regulation was detected in two individuals but also slight hyper-regulation was found in other two individuals. In any case, the capacity to hypo-osmoregulate must be clearly established at more advanced juvenile stages. Hypo-osmoregulation in adults was not found by Watanabe (1982) and not studied by Hudson *et al*. (2018); the latter study did not quantify osmoregulation at concentrated seawater. Differences between our finding and that of Watanabe (1982) may be due to either different temperatures used to acclimate crabs in the laboratory (this study = 24°C, Watanabe = 15°C), different range of salinities or genetic variation among populations. The pattern of hyper–hypo regulation is however consistent with that found in other species of the same superfamily (e.g. *Sesarma meinerti*: Gross *et al*., 1966; *S. curacaoense*: Anger and Charmantier, 2000; *A. miersii*: Charmantier *et al*., 1998; *N. granulata*: Charmantier *et al*., 2002).

Most larval stages of *H. sanguineus* (except the zoea V) had the capacity to osmoregulate over the range 15–25 ppt; this pattern and the high survival in response to acute exposure to low salinity (Table 1) are partially consistent with experiments reporting larval salinity tolerance (Epifanio *et al*., 1998). Such experiments also report a reduction in tolerance of the megalopa, but perhaps the reduction reflects carry-over effects from the zoea V, which showed a reduced capacity to osmoregulate. This ontogenetic pattern of osmoregulation of *H. sanguineus* appears to be intermediate between species showing retention strategy, where the OC is maintained or increased along the larval phase (e.g. Charmantier *et al*., 1998; Anger and Charmantier, 2000), and those showing export strategy, with osmo-conforming zoea II-IV stages (Charmantier *et al*., 2002; Cieluch *et al*., 2004; Anger *et al*., 2008). Laboratory experiments have shown that *H. sanguineus* larvae exhibit behaviours consistent with an export strategy (Cohen *et al*., 2015). However, the capacity to osmoregulate in the zoeal stages should provide *H. sanguineus* larvae opportunities to exploit estuarine habitats as no other known crab with such strategy. In those species, the timing of successfully crossing of salinity gradients should be constrained by the re-acquisition of the capacity to osmoregulate (e.g. see Torres *et al*., 2006) but our study shows that such constraint is weaker in *H. sanguineus*.

The pattern of osmoregulation in *H. sanguineus* compares well with the competitor, the European shore crab *Carcinus maenas*, also with an export strategy (Queiroga and Blanton, 2004). Larvae of both species share a trait that is critical for invasion in a context of global warming: that tolerance to low salinity increases with temperature (*C. maenas*: Spitzner *et al*., 2019; Torres, Thomas, Whiteley, Wilcockson, & Giménez, 2020, 2021a; *H. sanguineus*: Epifanio *et al*., 1998). However, *H. sanguineus* appear to have an advantage in three main points: First, the pattern of hyper–hypo regulation found in the adults for *H. sanguineus* is not present in *C. maenas*; hence, hypo-regulation may provide a competitive advantage to *H. sanguineus*, in intertidal habitats where salinity may increase over summer months at low tide, since it increases survival at high salinity through ion excretion. Second, most larval stages of *H. sanguineus* osmoregulate while most stages of *C. maenas* do not (zoea II-IV stages do not osmoregulate: Cieluch *et al*., 2004). Third, at high temperatures (e.g. 24°C), the capacity to osmoregulate of the zoea I is lower in *C. maenas* (e.g. zoea I: 55–60 mOsm kg^−1^: Torres *et al.* 2021a) as compared to *H. sanguineus* (~ 100 mOsm kg^−1^, this study). Thus, in temperate estuaries, at the time of the initiation of the migration from estuarine to open waters, first stage larvae of *H. sanguineus* are better equipped than those of *C. maenas*. The advantage of *C. maenas* over *H. sanguineus* might occur at low temperatures (≤ 18°C)*.*

In synthesis, we have found two critical traits that make *H. sanguineus* stand-up as compared with competitors (including the global invader *C. maenas*) and with other species with similar life-history strategy and habitat. First, adults are hyper-osmoregulators at low salinity and hypo-regulate at high salinity; this combination makes them a species well adapted to intertidal zones and especially for the use of tidal pools at latitudes where salinity can increase during the summer season. Second, the capacity to osmoregulate is present along most of the larval phase, which provides opportunities for use and invasion of estuarine waters.

## Authors’ contributions

GT, LG and GC conceived the experimental design. GT performed the larval rearing. GC performed the OC determinations. GT, LG and GC analysed the osmoregulation data. GT and LG wrote the first draft. All authors contributed to the writing of the manuscript and gave final approval for publication.

## Compliance with Ethical Standards

The research presented in this paper complies with the guidelines from the directives 2010/63/EU of the European parliament and of the Council of 22 September 2010 on the protection of animals used for scientific purposes.

## Data accessibility

All data for this paper will be available from PANGAEA Data Publisher https://www.pangaea.de.
